# Knowledge, attitudes, and practices related to COVID-19 pandemic among adult population in Sidama Regional State, Southern Ethiopia: A community based cross-sectional study

**DOI:** 10.1371/journal.pone.0246283

**Published:** 2021-01-29

**Authors:** Amanuel Yoseph, Alemu Tamiso, Amanuel Ejeso

**Affiliations:** 1 School of Public Health, College of Medicine and Health Sciences, Hawassa University, Hawassa, Ethiopia; 2 Department of Environmental Health, College of Medicine and Health Sciences, Hawassa University, Hawassa, Ethiopia; Ordu University, TURKEY

## Abstract

**Introduction:**

COVID-19 incidence is increasing and different measures have been adopted to control the spread of the pandemic in Ethiopia. Among these measures, enhancing the knowledge, positive attitudes, and proper practices of prevention measures about the disease is a basic strategy to control it. However, community compliance to control measures is largely dependent on their knowledge, attitudes, and practices (KAP) towards COVID-19.

**Objective:**

To assess the current level of KAP towards COVID-19 pandemic and predictors among the rural dwellers in Sidama regional state, Southern Ethiopia; 2020.

**Methods:**

This community-based prospective cross-sectional study was carried out from May 1–30, 2020 on a sample of 1,278 adult populations in Sidama regional state, Southern Ethiopia. A multi-stage sampling technique was used to choice the study participants. The data were collected using a structured interviewer-administered questionnaire. We have entered data using Epi data version 3.1 and all analyses were done using SPSS version 25. KAPs scores of study participants based on their independent variables were compared using Chi-square test, t-test or one-way analysis of variance (ANOVA) as required. Bi-variable and multivariable logistic regression analyses were used to identify factors associated with KAP. The important assumptions of the logistic regression model were checked to be satisfied. Adjusted odds ratios (AOR) with a 95% confidence interval (CI) were calculated to assess the existence and strength of associations.

**Results:**

From a total of 1,214 study participants, the overall attained knowledge, attitude and practice score about COVID-19 were 90%, 82.4% and 65%, respectively. Among these, 43.9%, 37.5%, and 24.4% of the study participants had demonstrated good knowledge, high attitude and proper practice, respectively. The mean knowledge scores were significantly different between sex, categories of marital status, educational levels, main occupation, and the monthly income quintiles of the study participants (p<0.05). Similarly, the mean attitude scores significantly varied across educational levels, main occupations and marital status (p<0.05). Based on multivariable logistic regression analysis, main occupation of the government employees, education level of diploma and above, highest and second highest wealth rank were positively associated with COVID-19 prevention and control practice.

**Conclusions:**

The majority of study participants had showed good knowledge and optimistic attitude toward COVID-19. But, the level of practice lower than that expected to maximize effective control measures. Further public education interventions and community sensitization campaigns are required for rural adult population in the Sidama regional state, Ethiopia.

## Introduction

The corona-virus disease pandemic is a continuing pandemic of coronavirus disease 2019 (abbreviated “COVID-19”) and caused by a novel corona virus. The epidemic was first proclaimed in Wuhan, China, in December 2019 [1].

Corona virus is mainly transmitted through respiratory droplets. It may also be possible to acquire infection by touching a surface or object that has the virus on it and then touching our mouth, nose, and probable eyes [2, 3].

The disease is highly contagious, and its major clinical manifestations include fever, dry cough, runny or stuffy nose, sneezing, sore throat, headache, body aches, fatigue, chills, and shortness of breath. The Centers for Disease Control and Prevention (CDC) reported that symptoms of corona virus may appear in as few as two days, or as long as 14 days after exposure [4, 5].

People with certain health conditions have a higher risk of severe complications if they acquire the infection. These health conditions includes Chronic Obstructive Pulmonary Disease (COPD) and asthma, heart conditions, immune system conditions such as HIV, cancer, obesity, diabetes, kidney disease, liver disease, pregnant women and old aged people [6].

The WHO declared that epidemic to be a Public Health Emergency of International Concern in January 2020, and announced it as a pandemic in March 2020. As of June 2020, greater than 10.1 million cases of COVID-19 pandemic have been reported in 213 nations and territories, resulting in nearly greater than 501,874 deaths. Greater than 5.2 million individuals have recovered, though there may be a likelihood of relapse or reinfection [7].

As of June 2020, greater than 5,570 cases of COVID-19 pandemic have been reported in Ethiopia and resulting in approximately 94 deaths. Greater than 2,015 individuals have recovered, though there may be an opportunity of relapse or reinfection in Ethiopia. Out of 5,570 cases, 89.6% of the cases are of Ethiopian nationals and 11.4% of the total positive cases belong to foreign nationals. Fortunately, to date, the case fatality rate is only 1.7% [7, 8].

Currently, Ethiopia is suffering from the critical phase of the COVID-19 thus; there is a fear of rapid raise of infection. To date, majority of the COVID-19 infection is limited to the high- income society where the infected persons have a direct or indirect relationship with the infected countries (China, Arab Emirate, Japanese, Belgium, and Dubai etc.) [9].

A huge disaster might happen in case the COVID-19 is entered into the people who live in overcrowded conditions. This population is real not aware of the socio-economic and health consequences of COVID-19 pandemic and even do not have adequate resources to control and prevent this infectious disease. Also, the late or undiagnosed COVID-19 cases might create a complex chain of an infected person [10].

The Ethiopian government is considered all types of preventive measures to halt transmission. Accordingly, all of the schools, colleges, Universities, company, and offices have been already locked down for the security of the nation. All the international and national conferences and meetings have been cancelled until further notice. The use of public transportation from region to region has been limited, to prevent the global spread of coronavirus infection [11, 12].

In addition, the federal ministry of health in Ethiopia did substantial efforts to prevent and control the pandemic through different measures. Public education is considered as one of the most relevant measures that can help in the prevention and control of infectious diseases [13].

Besides, above all the governmental efforts, people need to be aware of all government advisories, messages, guidelines and do follow the prevention measure for their safety and well-being [12]. Despite these efforts, nearly all people neglect the benefit of social distancing, hands washing, stay at home, use of sanitizer and mask due to attitudinal and behavioral issues.

The prevention and control measures against corona virus pandemic are proceeding in Ethiopia. To assure the final win against the battle, population compliance to these prevention and control measures are important, which is largely influenced by their knowledge, attitudes, and practices towards corona virus in agreement with KAP theory. In addition, to facilitate the prevention and control of COVID-19 in Ethiopia, there is an urgent need to measure the population awareness of COVID-19 at this critical time. Hence, it is relevant to study these domains in the Sidama adult population. Considering the importance of all the above factors, it was aimed to measure the current level of KAP towards COVID-19 pandemic and predictors among the adult population in rural Sidama regional state, Southern Ethiopia; 2020.

## Methods and materials

### Study area

This study was carried out in Sidama regional state, Southern Ethiopia. Sidama regional state is one of the 10th regions in Ethiopia and located 275 km south of Addis Ababa, the capital city of country. The region has consisted of six town administrations and thirty districts with an overall of 576 kebeles (lowest administrative units in Ethiopia with approximately 1,000 households). Based on the Ethiopian Population Census Report 2007 estimates, the overall population of the region in 2020 has reached 5,493,516.

The Sidama regional state has overall of 5,806 health professionals of diverse disciplines and 624 health posts, 127 health centers, two general hospital, 13 primary hospitals and 1 comprehensive and specialized hospital owned by the government. There are also thirty-two private medium and six non-governmental (NGO) clinics, and 73 private drug stores. The physical health service coverage of the region was 90.3%. The Hawassa city is the capital city of the Sidama regional state. The main crops grown in the area are enset (*false banana*), barely, khat, coffee, broad beans, cereals and livestock. Unimproved drinking water source, poor hygienic condition and frequent drought in the region results in high burden of the infectious diseases such as acute watery diarrhea and intestinal parasites and soil transmitted helminthiasis [14].

### Study design and population

This community-based cross-sectional study was carried out using a quantitative method in Sidama regional state from May 1–30; 2020. The source and study population of this study were all adult population and all systematically selected adult population who resided in the Sidama regional state for 6 months. The study participants who had lived less than 6 months, serious diseases, mental health disorders and treated last one month prior to survey for any illness were excluded from this study.

### Sample size determination

The minimum required sample size was estimated by using a single population proportion formula in consideration of the subsequent assumptions. The proportion (p) of KAP towards COVID-19 pandemic is 50% using a conservative approach due to the lack of previous study done in Ethiopia, 95% confidence level and 5% margin of error. As a multi-stage sampling technique was utilized to choice study participants, a design effect of 1.9 was taken and a 10% compensation for non-response was added to the estimated sample size. Thus, the final calculated sample size was 1,278.

### Sampling procedure

A multistage sampling technique was used to select the study participants. The region consists of 30 rural and 6 urban districts. Simple random sampling technique was used to select 9 out of 30 rural districts. Initially, the 30 rural district administrations (RDAs) of the region were clustered into three major sub-groups based on their proximity to Hawassa city, the capital of regional state. The first cluster includes 10 RDAs that are found south of the Daye city, the second cluster includes 10 RDAs that are found between Daye and Yirgalem city and the third cluster includes 10 RDAs that are found north of the Yirgalem city. From the ten RDAs that are found south of the Daye city, three RDAs (Chabe, Girga and Bensa) were selected randomly for the study. Also, from the ten RDAs that are found between Daye and Yirgalem, three RDAs (Bona, Titicha, and Chuko) were selected randomly. Similarly, from the 10 RDAs that are found north of Yirgalem city, 3 RDAs (Gorche, Yiraba and Darara) were selected randomly taking into consideration their homogeneity. In 3 clusters, a total of nine RDAs were included for the study. The RDAs were further categorized into three agro-climate regions (lowland, midland and highland). From each agro-climate regions, 1 kebele was selected purposively based on its information potential and accessibility. Firstly, the total calculated sample size was apportioned to the kebeles comparative to their population size. The calculated sample interval (K = N/n) was K (which was differ from kebele to kebele). Lastly, study respondents were drawn using a systematic sampling procedure with sampling interval of K. The first HH head was drawn by using simple random sampling procedure. Then, sequential HH head were selected at a regular interval of Kth household until required sample size obtained. If head of HH lacking from the household for three successive visits and there were no other choices, the subsequent nearest HH head was involved.

### Study variables

The dependent variables were KAP towards COVID-19 pandemic. The exposure variables were socio-demographic variables such as sex, age, religion, ethnicity, family size, monthly income quintiles, educational levels, occupation status, marital status and sources of information about the corona virus disease etc.

### Data collection tools and procedure

The data were collected using a properly designed, structured and pretested interviewer-administered questionnaire in Sidama language (the local language of the study region). The survey tool or questionnaire was prepared initially in English language, converted into Sidama language and then retranslated back to English language. The comparison was carried out to assess consistency and accuracy between the two versions of survey tools **(See S1 and S2 Files)**. Data collectors and supervisors were trained on the survey tool, data collection techniques, aims of the study, and ethical considerations by the research team for two days. Following the training, the survey tool was pre-tested on 5% of an overall calculated sample size among adult population in kebele which was not included in the actual study setting. At that instant, any inconsistency and non-accuracy documented between the two versions of survey tool were readjusted accordingly.

The data collection process was managed by 20 clinical Nurses. Four BSc Nurses were cautiously supervised the data collection method. The survey questionnaire was modified from standard the Question and Answer about COVID-19 in the website of WHO weekly report and other previous study conducted on KAP towards the Middle East Respiratory Syndrome Coronavirus (MERs CoV) [15, 16].

A structured study tool (questionnaire) comprised four parts. The first parts included socio-demographic and economic characteristics of the study respondents as aforementioned above in the study variables part. The second part comprised 12 questions about the knowledge of COVID-19. Knowledge questions were both in the form of a multiple response or in the arrangement of true, false, or no opinion (K1-K12). Correct responses had assigned 3 scores, whereas incorrect responses were assigned 1 score. A “no opinion” response had 2 scores. Finally, the overall knowledge point extended from 12 to 36. The study participants who had scored 31 and below were classified as having poor knowledge, 31 to 34 as having moderate knowledge, and above 34 as scoring high knowledge of COVID-19.

The third part assessed the attitude concerning COVID-19. To assess the attitude of the adult population towards the COVID-19, 19 questions were tested (A1-A19). The responding and scoring systems were the same to the second part which was concerning knowledge (correct = 3, incorrect = 1, and no opinion = 2). The overall attitude point extended from 19 to 57. A score of below 47, 47 to 50, and above 50 was categorized as low, moderate, and high attitude towards COVID-19, respectively.

The last part included 11 questions regarding the practice of COVID-19. Concerning the adult population's practice towards the COVID-19, 11 questions were tested (P1-P11), with the same grading system as earlier (true = 3, false = 1, no opinion = 2). Overall scores of below 21, 21 to 24, and above 24 were categorized as a weak, moderate, and strong practice towards COVID-19, respectively.

This paper was prepared based on the Strengthening the Reporting of Observational studies in Epidemiology (STROBE) guideline and the STROBE checklist is delivered as supporting information **(See S3 File).**

### Data analysis procedure

Data were entered into Epi data version 3.1 and all analyses was done using SPSS version 25. All needed variables recoding and calculations were carried out before to the main analysis. The income quintile of the study participants used as a measure of the socioeconomic status of the study participants. The descriptive analyses were done to find out descriptive measures for the socio-demographic and other important variables. The knowledge, attitudes and practices scores of study participants based on their independent variables were compared using Chi-square test, t-test, one-way analysis of variance (ANOVA) as required.

The Spearman’s correlation analysis was used to measure the association among mean attitude and practice scores. Data were analyzed using bi-variable and multivariable logistic regression model. The variables with p-values < 0.25 on the bi-variable analysis were entered into a multivariable binary logistic regression model to find out predictors independently associated with KAP adjusting for other predictors in the model. The candidate variables were entered into the multivariable binary logistic regression model using the backward (Wald) stepwise regression technique. The basic assumptions of binary logistic regression model such as absence of outliers, multicollinearity and interaction between independent variables were tested to be fulfilled. The multicollinearity among the exposure variables was also evaluated using multiple linear regression model. For all variables as the variance inflation factor (VIF) was less than 10 which indicates no evidence of multicollinearity. Fitness of binary logistic regression model was considered using the Hosmer-Lemeshow statistics in model and greater than 0.05. The existence and strength of association between KAP and the exposure variables were evaluated using adjusted odds ratios (AORs) with 95% confidence intervals. The statistically significant association between the variables of interest was confirmed when the 95% CI of the AOR did not embrace 1.

### Ethics statement

Ethical clearance was got from the Hawassa University, institutional review board with a reference number of IRB/143/10. The informed written consent was obtained from the sampled study participants. The study participants with symptom and sign of COVID-19 were referred to nearby isolation centers for further investigation and treatment. The ethical considerations were addressed by proving the cloth face mask, sanitizer, alcohol and soap for the study participants.

## Results

Socio-demographic and economic characteristics of the respondents have been summarized in Table 1. From a total of 1,278, merely 1,214 study participants responded questions, making a response rate of 95%. The mean ± standard deviation [SD]) of the age of respondents was 34 ±10) years and (ranged, 18–80). Majority of respondents were within the range of 21–30 years and male 710 (58.5%). The mean family size of study household was 5 persons. According to this study, majority 1161/1214 (95.6) of the study participants were from Sidama ethnic group. Majority 1161/1214 (95.6%) and 1,024/1,214 (84.3%) of the study participants were followers of protestant Christianity and married, respectively. Majority, 977/1,214 (80.4%) of the study participants joined formal education. Almost all 1,206/1,214 (99.3%) of the studied households had access to news media such as TV and radio.

**Table 1:** Socio-demographic and economic characteristics of the study participants in rural Sidama regional state, Southern Ethiopia, 2020 (N = 1214).

Variables	N (%)
**Sex**	
Male	710 (58.5)
Female	504 (41.5)
**Age (years)**	
< 20	111 (9.1)
21–30	503 (41.4)
31–40	371 (30.6)
41–50	148 (12.2)
> 50	81 (6.7)
**Marital status**	
Single	128 (10.5)
Married	1024 (84.3)
Divorced	30 (2.5)
Widowed	32 (2.6)
**Religions**	
Protestant	1141 (94.0)
Orthodox	38 (3.1)
Catholic	16 (1.3)
Muslim	19 (1.6)
**Ethnicity**	
Sidama	1161 (95.6)
Amhara	36 (3.0)
Oromo	4 (0.3)
Gurage	8 (0.7)
Wolayita	5 (0.4)
**Education status**	
Cannot read and write	178 (14.7)
Read and write only	59 (4.9)
Primary education (1–8)	531 (43.7)
Secondary education (9–12)	243 (20.0)
Diploma and above	203 (16.7)
**The main occupation of the participants**	
Student	104 (8.6)
Merchant	292 (24.1)
Employee	190 (15.7)
Farmer	357 (29.4)
Daily laborer	29 (2.4)
House wife	242 (19.8)
**Family size**	
Small	890 (73.3)
Medium	220 (18.1)
Large	104 (8.6)
**Monthly income quintiles**	
Lowest	425 (35.0)
Second lowest	55 (4.5)
Middle	248 (20.4)
Second highest	217 (17.9)
Highest	269 (22.2)

### Knowledge of the study participants towards COVID-19

The majority of study participants knew that cause of COVID-19 is a virus, symptom such as fever, cough, sore throat, shortness of breath, and patients with certain conditions are at a higher risk for COVID-19 infection and death (73.0%, 83.0%, and 69.0%, respectively). Similarly, almost all of study participants knew that COVID-19 can be prevented through different mechanisms, the isolation of suspected cases should be for a minimum of 2–14 days, and vaccine were not available in market (98.9%, 60.5%, and 67.9%, respectively). According to our results, the majority, 533 (43.9%) of the adult population had good knowledge about the disease. The mean knowledge score was 32.4, indicating an overall 90% correct rate for knowledge questions (Table 2). Based on t-test and ANOVA analysis, the mean knowledge scores were significantly different among sex, categories of marital status, educational levels, main occupation, and the monthly income quintiles of the study participants. Government employees showed higher knowledge scores (33.7 ± 3.5) than daily laborer (31.5 ± 3.9) and house wife (30.9 ± 4) (p<0.05) (Table 3).

**Table 2:** Knowledge of study participants toward COVID-19 in rural Sidama regional state, Southern Ethiopia, 2020 (N = 1214).

Item (correct response)	N (%)
Do you hear about the COVID-19 (yes)	1206 (99.3)
Cause of COVID-19 is virus infection (yes)	886 (73.0)
Is COVID-19 a transmissible/contagious disease (yes)	771 (71.4)
Fever, cough, sore throat, shortness of breath are symptom of COVID-19 (yes)	1008 (83.0)
The isolation period is 2–14 days (yes)	735 (60.5)
Patients with certain conditions are at a higher risk for COVID-19 infection and death (yes)	838 (69.0)
The prevalence of COVID-19 disease is increasing in Ethiopia (yes)	847 (69.8)
COVID-19 vaccine is present in market (no)	824 (67.9)
COVID-19 can be transmitted from household pets to humans (no)	1010 (83.2)
COVID-19 treatment is available in market (no)	885 (72.9)
Can COVID-19 be prevented (yes)	1201 (98.9)
Can COVID-19 results in death in all cases (no)	1188 (97.9)
Knowledge toward COVID-19 (mean + SD)	(32.4+ 3.9)
Knowledge toward COVID-19	
Poor	422 (34.8)
Moderate	259 (21.3)
Good	533 (43.9)

**Table 3:** Distribution of knowledge scores among adult population in Sidama regional state, Southern Ethiopia, 2020.

**Variables**	**N (%)**	**Knowledge score**	***t/F*-value**	***P*-value**
**Age**			0.3	0.87
≤ 20	111 (9.1)	32.5 + 4.0		
21–30	503 (41.4)	32.5 + 3.9		
31–40	371 (30.6)	32.3 + 4		
41–50	148 (12.2)	32.4 + 3.8		
≥ 50	81 (6.7)	32.2 + 3.6		
**Sex**			19.66	0.001**
Male	710	33.8 + 3.7		
Female	504	30.8 + 4		
**Marital status**			3.3	0.02*
Single	128	32.5 + 4.2		
Married	1024	32.4 + 3.8		
Divorced	30	30.2 + 4		
Widowed	32	31.9 + 4.3		
**Educational status**			13.4	0.03*
Cannot read and write	178	31.1 + 3.6		
Read and write only	59	31.4 + 3.8		
Primary education (1–8)	531	32.2 + 4		
Secondary education	243	33 + 3.7		
Diploma and above	203	33.6 + 3.6		
**Monthly income quintiles**			10.9	0.001**
Lowest	425 (35.0)	31.7 + 4		
Second lowest	55 (4.5)	31.4 + 4.4		
Middle	248 (20.4)	32.4 + 3.7		
Second highest	217 (17.9)	32.6 + 3.8		
Highest	269 (22.2)	33.7 + 3.4		
**The main occupation of the participants**			12.4	0.001**
Farmer	357 (29.4)	31.3 + 3.7		
Student	104 (8.6)	32.8 + 4		
Merchant	292 (24.1)	32.8 + 3.8		
Employee	190 (15.7)	34.7 + 3.5		
Daily laborer	29 (2.3)	31.5 + 3.9		
House wife	242 (19.9)	30.9 + 4		

Data were described as mean±SD. t-test and ANOVA were test used to a comparison between socio-demographic characteristics of study participants and the score of knowledge.

*: Indicates significant association (P < 0.05)

**: Indicate the highly significant association (P <0.01).

### Attitude of the study participants towards COVID-19

The majority, 1009 (83.1%) of the study participants had afraid to go to common places in order to avoid infection and took precautions 774 (63.8%) to prevent infection. More than three-fourth of the study participants had confidence that Ethiopia government able to control the pandemic, while 23.5% had no such confidence. Only 14.4% of the study participants had reported that COVID-19 has no implication on Ethiopian economy. The mean (+standard deviation [SD]) correct response score of the 19 questions about the attitude towards COVID-19 rate was 47 (+5), indicating an overall 82.5% correct rate on this attitude test. The majority, 508 (47.8%) of the study participants had a low attitude towards COVID-19 (Table 4). The mean attitude scores significantly varied across educational levels, main occupations and marital status (p<0.05). Study participants who were in secondary education level showed higher attitude scores (47.9 ± 4.7) than primary education level (47.1 ± 5.2) and cannot read and write (46.1 ± 5.3) (Table 5).

**Table 4:** Attitude of study participants toward COVID-19 in rural Sidama regional state, Southern Ethiopia, 2020 (N = 1214).

Item (response)	N (%)
Do you think that disease is dangerous (yes)	1177 (97.0)
Do you think that COVID-19 is curse of God (no)	243 (20.0)
Are you worried about one of your family members can get infection (yes)	601 (49.5)
Are you afraid to go to common places in order to avoid infection (yes)	1009 (83.1)
Do you think the early diagnosis improves the treatment and outcome (yes)	847 (69.8)
Do you think the isolation of the suspected cases is important (yes)	1010 (83.2)
Do you think health education is important to prevent COVID-19 (yes)	1188 (97.9)
If you take precautions, can the COVID-19 infection be prevented (yes)	774 (63.8)
If you know that the animals are sources for the transmission of COVID-19, would you consume raw animals’ milk or meat (no)	712 (58.6)
If there is a vaccine, would you take it (yes)	1034 (85.2)
Can COVID-19 about infection be cured (yes)	865 (71.3)
Is the available information about COVID-19 in Ethiopia sufficient (yes)	870 (71.7)
Are the protective measures sufficient for prevention (yes)	679 (55.9)
Is there negative effect of infection on Ethiopian economy (yes)	1039 (85.6)
Does the government institutions able to control the pandemic (yes)	929 (76.5)
Do you think yourself at risk (yes)	825 (68.0)
If you have one of the symptoms of the disease do you go to the health facility (yes)	627 (51.6)
If you have COVID-19 symptoms, do you avoid normal activities (yes)	915 (75.4)
Do you avoid contact with infected case (yes)	1203 (99.1)
Attitude towards COVID-19 (mean + SD)	(47 + 5)
Low	580 (47.8)
Moderate	179 (14.7)
High	455 (37.5)

**Table 5:** Distribution of attitude scores among adult population in Sidama regional state, Southern Ethiopia, 2020.

**Variables**	**N (%)**	**Attitude score**	**t/F-value**	***P*-value**
**Age**			0.3	0.85
≤ 20	111 (9.1)	47.2 + 5.0		
21–30	503 (41.4)	46.9 + 5.0		
31–40	371 (30.6)	47.3 + 5.2		
41–50	148 (12.2)	47.1 + 4.9		
≥ 50	81 (6.7)	47.3 + 4.8		
**Sex**			0.2	0.65
Male	710	47.1 + 5.2		
Female	504	47.2 + 5.1		
**Marital status**			3.1	0.026*
Single	128	46.6 + 5.3		
Married	1024	47.3 + 5.0		
Divorced	30	44.9 + 5.6		
Widowed	32	46.0 + 6.2		
**Educational status**			3.6	0.007*
Cannot read and write	178	46.1 + 5.3		
Read and write only	59	46.1 + 5.5		
Primary education (1–8)	531	47.1 + 5.2		
Secondary education	243	47.9 + 4.7		
Diploma and above	203	47.5 + 5.2		
**Monthly income quintiles**			1.21	0.302
Lowest	425 (35.0)	47.4 + 5.1		
Second lowest	55 (4.5)	46.5 + 6.2		
Middle	248 (20.4)	46.8 + 5.4		
Second highest	217 (17.9)	47.4 + 4.9		
Highest	269 (22.2)	46.8 + 4.9		
**The main occupation of the participants**			4.23	0.001**
Farmer	357 (29.4)	45.9 + 5.0		
Student	104 (8.6)	47.1 + 5.0		
Merchant	292 (24.1)	46.4 + 5.2		
Government employee	190 (15.7)	49.1 + 5.0		
Daily laborer	29 (2.3)	46.2 + 5.7		
House wife	242 (19.9)	46.6 + 5.4		

Data were described as mean ± SD. t-test and ANOVA were test used to a comparison between socio-demographic characteristics of study participants and the score of attitude.

*: Indicates significant association (P < 0.05)

**: Indicate the highly significant association (P <0.01).

### Practice of the study participants towards COVID-19

The majority of study participants had washed hands often with soap, avoided shaking hands and stayed at home to prevent COVID-19 infection (93.0%, 81.0%, and 80.9%, respectively). The mean (+standard deviation [SD]) correct response score of the 11 questions concerning the rate of the COVID-19 practice was 21.5 (+ 3.8), indicating an overall 65% correct rate in the practices test. The majority, 542 (44.6%) of study participants had weak practice towards COVID-19 (Table 6). The Spearman’s analysis showed that a significant positive correlation among the mean attitude and practice scores regarding COVID-19 (r = -0.61, P<0.001). The higher the attitude scores were, the higher the probability of positive practices. Hence, a positive attitude toward COVID-19 was directly associated with a positive practice. The mean practice scores significantly varied across sex, marital status, education levels, monthly income quintiles, and the main occupation of the study participants. Study participants who were in highest wealth rank showed higher practice scores (22.4 ± 3.6) than lowest (20.6 ± 3.6) and middle rank (21.6 ± 3.8) (Table 7).

**Table 6:** Practice of study participants toward COVID-19 in rural Sidama regional state, Southern Ethiopia, 2020 (N = 1214).

Variables (response)	N (%)
I wash hands often with soap (yes)	1165 (96.0)
I avoid touching the eyes, noses and mouth with unwashed hands (yes)	359 (29.6)
I throw the tissue in the trash after I use (yes)	216 (17.8)
I use mask to cover my nose and mouth in crowded places (yes)	814 (67.1)
I stay at home (yes)	982 (80.9)
I maintain social distance (yes)	690 (56.8)
I disinfect frequently touched surfaces and objects (yes)	148 (12.2)
I use face mask (yes)	814 (67.1)
I avoid shaking hands (yes)	983 (81.0)
I use herbal medicine (yes)	878 (72.3)
I use antibiotics (yes)	476 (39.2)
Practice towards COVID-19 (mean + SD)	(21.5 + 3.8)
Weak	542 (44.6)
Moderate	376 (31.0)
Strong	296 (24.4)

**Table 7:** Distribution of practice scores among adult population in Sidama regional state, Southern Ethiopia, 2020.

**Variables**	**N (%)**	**Practice score**	**t/F-value**	***P*-value**
**Age**			2.48	0.053
≤ 20	111 (9.1)	21.3 + 3.7		
21–30	503 (41.4)	21.8 + 3.7		
31–40	371 (30.6)	21.2 + 4.0		
41–50	148 (12.2)	21.1 + 3.6		
≥ 50	81 (6.7)	21.9 + 3.6		
**Sex**			16.43	0.003*
Male	710	21.9 + 3.8		
Female	504	21.0 + 3.7		
**Marital status**			8.07	0.001**
Single	128	22.5 + 3.9		
Married	1024	21.3 + 3.7		
Divorced	30	23.7 + 2.5		
Widowed	32	22.3 + 5.1		
**Educational status**			21.88	0.005*
Cannot read and write	178	21.3 + 4.0		
Read and write only	59	22.3 + 3.8		
Primary education (1–8)	531	21.0 + 3.6		
Secondary education	243	21.6 + 3.5		
Diploma and above	203	23.4 + 3.6		
**Monthly income quintiles**			15.38	0.001**
Lowest	425 (35.0)	20.6 + 3.6		
Second lowest	55 (4.5)	20.0 + 4.3		
Middle	248 (20.4)	21.6 + 3.8		
Second highest	217 (17.9)	22.3 + 3.7		
Highest	269 (22.2)	22.4 + 3.6		
**The main occupation of the participants**			10.22	0.001**
Farmer	357 (29.4)	20.2 + 3.6		
Student	104 (8.6)	21.7 + 3.9		
Merchant	292 (24.1)	21.2 + 3.8		
Employee	190 (15.7)	24.2 + 3.7		
Daily laborer	29 (2.3)	21.1 + 3.7		
House wife	242 (19.9)	20.8 + 3.7		

Data were described as mean ± SD. t-test and ANOVA were test used to a comparison between socio-demographic characteristics of study participants and the score of practice.

*: Indicates significant association (P < 0.05)

**: Indicate the highly significant association (P <0.01).

Results of the bi-variable and multivariable logistic regression analysis of knowledge towards COVID-19 are shown in Table 8. Multivariable analysis of predictors revealed that odds of knowledge towards COVID-19 infections were 2.51 times increased in males sex as compared to those females (AOR = 2.51; 95% CI = 1.65–3.78; P = 0.001). The secondary education levels (AOR = 2.38; 95% CI = 1.50–3.78; P = 0.001) and occupation status of government employees (AOR = 3.18; 95% CI = 1.92–5.02; P = 0.008) were positively associated with knowledge towards COVID-19 infections. The odds of knowledge towards COVID-19 infections were 2.41 times increased for the study participants who had a diploma and above educational level (AOR = 2.41, 95 CI = 1.31–4.43) as compared to those who had cannot read and write (Table 8).

**Table 8:** Bi-variable and multivariable regression of association between socio-demographic and economic characteristics with the knowledge scores of COVID-19 among adult population in Sidama regional state, South Ethiopia, 2020.

Variables	Knowledge		COR	AOR
	Insufficient	Sufficient		
**Age**				
< 20	45 (40.5)	66 (59.5)	1.66 (1.03, 2.95)	1.44 (0.69, 3.02)
21–30	207 (41.2)	296 (58.8)	1.61 (1.01, 2.59)	1.34 (0.76, 2.35)
31–40	158 (42.6)	213 (57.4)	1.52 (0.94, 2.47)	1.36 (0.80, 2.33)
41–50	64 (43.2)	84 (56.8)	1.48 (0.86, 2.55)	1.35 (0.76, 2.39)
> 50	43 (53.1)	38 (46.9)	1	1
**Sex**				
Male	219 (30.8)	491 (69.2)	3.24 (2.26, 5.01)	2.51 (1.65, 3.78)**
Female	298 (59.1)	206 (40.9)	1	1
**Marital status**				
Single	49 (38.3)	79 (61.7)	1.61 (1.02, 3.51)	0.96 (0.38, 2.38)
Married	430 (42.0)	594 (58.0)	1.38 (0.68, 2.79)	1.12 (0.51, 2.45)
Divorced	22 (73.3)	8 (26.7)	0.36 (0.12, 1.05)	0.26 (0.08, 0.83)
Widowed	16 (50)	16 (50)	1	1
**Educational status**				
Cannot read and write	106 (59.6)	72 (40.4)	1	1
Read and write only	35 (59.3)	24 (40.7)	1.01 (0.55, 1.83)	0.89 (0.47, 1.66)
Primary education (1–8)	238 (44.8)	293 (55.2)	1.81 (1.28, 2.55)	1.57 (1.06, 2.30)*
Secondary education (9–12)	84 (34.6)	159 (65.4)	2.78 (1.86, 4.15)	2.38 (1.50, 3.78)*
Diploma and above	54 (26.6)	149 (73.4)	4.06 (2.63, 6.25)	2.41 (1.31, 4.43)**
**Monthly income quintiles**				
Lowest	207 (48.7)	218 (51.3)	1	1
Second lowest	26 (47.3)	29 (52.7)	1.05 (0.60, 1.85)	1.55 (0.68, 3.50)
Middle	110 (44.4)	138 (55.6)	1.19 (0.87, 1.63)	2.01 (0.77, 5.25)
Second highest	95 (43.8)	122 (56.2)	1.21 (0.88, 1.69)	2.25 (0.52, 6.73)
Highest	79 (29.4)	190 (70.6)	2.28 (1.65, 3.15)	5.02 (0.67, 8.82)
**The main occupation**				
Student	41 (39.4)	63 (60.6)	2.07 (1.29, 3.31)	1.52 (0.84, 2.75)
Merchant	116 (39.7)	176 (60.3)	2.04 (1.44, 2.89)	1.38 (0.91, 2.07)
Employee	40 (21.0)	150 (79.0)	5.06 (3.03, 7.86)	3.18 (1.92, 5.02)**
Farmer	155 (43.4)	202 (56.6)	1.75 (1.26, 2.44)	0.63 (0.27, 1.45)
Daily laborer	17 (58.6)	12 (41.4)	0.95 (0.43, 2.08)	0.99 (0.91, 1.01)
House wife	139 (57.4)	103 (42.6)	1	1

1: Indicates the reference categories

*: Indicates significant association (P-value < 0.05)

**: Indicate the highly significant association (P-value <0.01).

Both bi-variable and multivariable analyses of predictors revealed that odds of attitude towards COVID-19 were 3.07 times higher in study participants who have good knowledge as compared to those who have poor knowledge about COVID-19 (AOR = 3.07; 95% CI = 2.79–5.04; P = 0.004). In the multivariable model in which 7 potential confounders were adjusted, the odds of knowledge towards COVID-19 were 3.16 times increased (AOR = 3.16, 95% CI: 2.71–5.89) among study participants who had government employees as compared to the students (Table 9).

**Table 9:** Bi-variable and multivariable regression of association between socio-demographic and economic characteristics with the attitude scores of COVID-19 among adult population in Sidama regional state, Southern Ethiopia, 2020.

Variables	Attitude		COR	AOR
	Negative	Positive		
**The main occupation**				
Student	54 (51.9)	50 (48.1)	1	1
Merchant	157 (53.8)	135 (46.2)	0.92 (0.59, 1.45)	0.95 (0.60, 1.49)
Employee	39 (20.5)	151 (79.5)	4.18 (2.75, 7.77)	3.16 (2.71, 5.89)**
Farmer	188 (52.7)	169 (47.3)	1.03 (0.80, 2.66)	0.98 (0.65, 1.93)
Daily laborer	16 (55.2)	13 (44.8)	0.87 (0.38, 2.00)	0.95 (0.41, 2.19)
House wife	126 (52.1)	116 (47.9)	0.99 (0.62, 1.57)	1.14 (0.71, 1.82)
**Knowledge toward COVID-19**				
Poor	218 (51.7)	204 (48.3)	1	1
Moderate	144 (55.6)	115 (44.4)	0.85 (0.62, 1.16)	0.80 (0.58, 1.10)
Good	118 (40.9)	415 (59.1)	6.66 (3.19, 8.99)	3.07 (2.79, 5.04)*

1: Indicates the reference categories

*: Indicates significant association (P-value < 0.05)

**: Indicate the highly significant association (P-value <0.01).

Multivariable analysis of predictors revealed that odds of practice towards COVID-19 prevention and control were 2.56 times higher in respondent who had married as compared to those who had single in marital status (AOR = 2.56; 95% CI = 1.54–4.16; P = 0.004). In addition, main occupation of the government employee (AOR = 3.01; 95% CI = 2.44–5.39) and education level of diploma and above (AOR = 2.48; 95% CI = 3.13–21.30) were positively associated with COVID-19 prevention and control practice. Moreover, the odds of practicing COVID-19 prevention and control were 2.62 times increased for respondent who had a highest wealth rank (AOR = 2.62, 95% CI = 1.81–3.81) as compared to those who had the lowest wealth rank (Table 10).

**Table 10:** Bi-variable and multivariable regression of association between socio-demographic and economic characteristics with the practice scores of COVID-19 among adult population in Sidama regional state, Southern Ethiopia, 2020.

Variables	Practice		COR	AOR
	Poor	Good		
**Marital status**				
Single	37 (28.9)	91 (71.1)	1	1
Married	490 (47.9)	534 (52.1)	2.27 (1.51, 3.44)	2.56 (1.54, 4.16)*
Divorced	6 (20)	24 (80)	2.64 (0.86, 8.09)	3.42 (1.05, 9.20)*
Widowed	11 (34.4)	21 (65.6)	0.77 (0.34, 1.76)	0.73 (0.29, 1.84)
**Educational status**				
Cannot read and write	99 (55.6)	79 (44.4)	1	1
Read and write only	18 (30.5)	41 (69.5)	2.85 (1.52, 5.34)	1.51 (0.99, 4.83)
Primary education (1–8)	266 (50.1)	265 (49.9)	1.24 (0.88, 1.75)	1.02 (0.70, 1.48)
Secondary education	107 (44.0)	136 (56.0)	1.59 (1.07, 2.35)	1.18 (0.76, 1.84)
Diploma and above	52 (25.6)	151 (74.4)	3.63 (2.36, 5.60)	2.48 (1.36, 4.52)**
**Monthly income quintiles**				
Lowest	236 (55.5)	189 (44.5)	1	1
Second lowest	33 (60.0)	22 (40.0)	0.83 (0.47, 1.47)	0.83 (0.45, 1.52)
Middle	107 (43.1)	141 (56.9)	1.64 (1.20, 2.25)	1.97 (1.40, 2.77)*
Second highest	75 (34.6)	142 (65.4)	2.36 (1.68, 3.31)	2.42 (1.66, 3.54)*
Highest	91 (33.8)	178 (66.2)	2.44 (1.77, 3.35)	2.62 (1.81, 3.81)**
**Main occupation**				
Student	39 (37.5)	65 (62.5)	1.87 (1.16, 2.99)	1.01 (0.57, 1.81)
Merchant	133 (45.5)	159 (54.5)	1.34 (0.95, 1.88)	0.89 (0.61, 1.30)
Employee	38 (30.5)	152 (69.5)	4.49 (2.71, 6.80)	3.01 (2.44, 5.39)**
Farmer	187 (46.8)	170 (53.2)	1.02 (0.92, 1.77)	0.72 (0.62, 2.01)
Daily laborer	17 (58.6)	12 (41.4)	0.79 (0.36, 1.73)	0.74 (0.33, 1.68)
House wife	128 (52.9)	114 (47.1)	1	1

1: Indicates the reference categories

*: Indicates significant association (P-value < 0.05)

**: Indicate the highly significant association (P-value <0.01).

## Discussion

To the best of our evidence, this is the first study carried out in Sidama regional state to assess the KAP towards COVID-19 among the general adult population. Based on our results, the KAP towards COVID-19 score was significantly higher among males, highest wealth rank, married people, those who had higher education levels and government employees.

According to the knowledge scores of the study respondents, an overall correct rate of 90% for the knowledge questions, verified that the majority of study respondents were good knowledge towards COVID-19 pandemic. This result is consistent with the previous studies findings from China and Iran general population concerning the KAP towards COVID-19; however, a poor knowledge about the isolation period of disease and patients with certain conditions are at a higher risk for COVID-19 infection and death was demonstrated among the general adult population of Sidama region [17, 18]. This high correct answer rate concerning knowledge toward COVID-19 among Sidama population might be due to the fact that its origins somewhat in their high exposure to the information delivered by the Ethiopian government and different media about the virus from the time when the start of the epidemic. Another reason might be the fact that study participants answered energetically to the severe circumstance of the pandemic and the enormous news reports, by gathering information from honorable sources.

In our study the KAP towards COVID-19 are higher as compared to a study conducted in Northern Thailand about KAP towards COVID-19 among the bordered population in the early period of the epidemic [19]. Majority, 73.4% of the study participants had poor knowledge of COVID-19 prevention and control in Thailand; whereas in our study only 34.8% had demonstrated poor knowledge score. Also, 28.5% had high attitude towards COVID-19 prevention and control mechanisms; however, in this study 37.5% had high attitude towards COVID-19.

In this study the proper/excellent skills to prevent and control the pandemic was 24.4%. In opposite to the current finding, studies conducted in Thailand among the bordered population in the early period of the epidemic reported a lower proper practice score (113.6%) [19]. This difference might be due to the fact that variation in the sample size, study population, area, and period. In Sidama region, the study was conducted at the time of the key phase of the pandemic after the population were wide-open to a lot of information about the COVID-19, its sign and symptom, isolation period, ways of transmission and prevention mechanisms, in Thailand the study was carried out in the early period of the outbreak.

However, the studies conducted in Iran and China reported higher KAP scores towards COVID-19 than our study [17, 18]. This difference might be due to the fact that Iran and China surveys included urban study respondents. But, our study included study respondents from rural area. In addition, another reason might be due to the fact that 75.1% and 82.4% of the study respondents held an academic degree and associate’s degree and above in Iran and China, respectively.

This study found that good knowledge score about COVID-19 was positively associated with a positive attitude and good practice towards disease at the time of pandemic. This finding signify the importance of improving general public knowledge’s about disease by public education program through the different channels which, in turn, would increase their positive attitude and good practice about COVID-19. This finding is in agreement with the studies done in

Our results of the socio-demographic and economic characteristics associated with KAP regarding COVID-19 are almost the same to earlier KAP studies about SARS and COVID-19 in China [18, 20]. According to these results, in order to improve public education support programs concerning the knowledge about COVID-19, more directed/focused methods for certain socio-demographic and economic groups such as female sex, lowest and second lowest wealth rank, farmer, house wife and those with lower education status are required.

The overall attitude score towards disease was 82.5% in the study area among the adult Sidama population. This result was significantly lower among study respondents who have poor knowledge scores, divorced and widowed, lower educational levels, daily laborer and house wife. Based on this study results, the majority of the study participants agreed with providing health education is important to prevent COVID-19, to take vaccine if there is a vaccine, available information about COVID-19 in Ethiopia is sufficient and to avoid normal activities if they have one of the symptom of COVID-19 (97.9%, 85.2%, 71.7% and 75.4%, respectively).

Earlier experience from the SARS virus epidemic revealed that all-embracing implementation of traditional public health measures in the lack of vaccine and antiviral management totally halt person-to-person transmission and the virus was final successful eradicated. In this closely associated virus, public adherences for such measures are important to prevent the human-to-human transmission of COVID-19 by separating population to halt spread and to control the outbreak of disease. The firearm tools we have at hand are public adherence to the preventive measure such as frequent hands washing with soap, isolation of the suspected cases and quarantine, maintain social distance, use of mask in the crowded place and disinfecting frequently touched surface. All these important measures are presently being practiced at an unprecedentedly large scale in urban part [21].

Multivariable analyses of predictors revealed that odds of practice towards COVID-19 were 1.57 times higher in study participants who have positive attitude as compared to those who have negative attitude about COVID-19 (AOR = 1.57; 95% CI = 1.20–2.04; P = 0.004). However, in spite of this positive association between higher attitudes with higher practice in current study, only 81.0%, 80.9%, 67.1% and 56.8% of the study respondents avoid shaking hands, stayed at their home, use cloth face mask and maintain social distance, respectively. Additional establishment and reinforcement from the Ethiopian government is needed for the converting of these positive attitudes into proper practice.

### Limitation of the study

Our study had several strengths. From these, the community-based nature of this study is representative of all adult population which is vital to develop important policy strategy for effective prevention and control of COVID-19 pandemic. In the fact that we enrolled in a comparatively large number of study participants (n = 1,214) from different kebeles and presented proportion figures for knowledge, attitude and practice. Moreover, we tried to quantify and accounted for several potential confounders that can individually explain the association among the variables of interest. Irrespective of its strengths, this study has some fundamental limitations that might be careful while interpreting the results. Firstly, the cross-sectional nature of the study design does not precisely establish the cause and effect relationship. Secondly, the study might be liable to recall bias because, all part of the information was collected by the study participants self-report. Thirdly, there was limited studies conducted sufficiently to compare our result and the study samples were collected at the Sidama regional state in Southern Ethiopia in the present condition. Thus, it was challenging to generalize our findings across the general adult population. Upcoming studies could estimate the knowledge, attitude and practice of general adult population on a larger scale to be able to design proper interventions on a nationwide level.

## Conclusions

The results indicated that the majority of the study participants in rural Sidama regional state had demonstrated good knowledge, positive attitude and reasonable practice regarding COVID-19, however there are some negative attitudes and improper practice than expected to prevent and control the pandemic. Supplementary public education intervention and sensitization campaigns are needed for the study participants according to different factors (sex, marital status, occupation, educational levels and wealth status). Moreover, according to the significant positive association among knowledge, attitude, and practice in current study, public education programs, specifically aiming lower knowledge and negative attitude individuals about COVID-19, are vital for maintain proper practices. Confidently, by enhancing good knowledge and positive attitude through public health decision-makers, and the support of the Ethiopian government and the overall population, hopeful control and elimination of the COVID-19 can be expected.

## Supporting information

S1 File English version survey questionnaire. (DOCX)

S2 File Sidamic version survey questionnaire. (DOCX)

S3 File STROBE statement. (DOC)

S4 File Raw SPSS dataset. (SAV)

## List of abbreviations

ANOVA: Analysis of variance
AOR: Adjusted odd ratio
CDC: Centers for Disease Control and prevention
CI: Confidence Interval
COPD: Chronic Obstructive Pulmonary Disease
COVID-19: Corona virus disease 2019
KAP: Knowledge, attitude and practice
OR: Odd ratios
SD: Standard Deviation
SPSS: Statistical packages for social science
VIF: Variance inflation factors
WHO: World Health Organization
