# Supplementary material for: Knowledge, attitudes, and practices related to COVID-19 pandemic among adult population in Sidama Regional State, Southern Ethiopia: A community based cross-sectional study
Source: PLoS One. 2021 Jan 29;16(1):e0246283. doi: 10.1371/journal.pone.0246283 (PMC7846007; doi:10.1371/journal.pone.0246283)
Supplement: S1 File — (DOCX) [file pone.0246283.s001.docx]

# Annex I: English version questionnaire

**Hawassa University**

**College of Medicine and Health Sciences**

**School of Public Health**

***Questionnaire designed to study the general public KAP towards COVID-19 among the adult population of Sidama regional state***

**1. Information sheet**

Hello! My name is _------------------------------------------------------_. I am a data collector in a survey being conducted about COVID-19 under the College of Medicine and Health Sciences of Hawassa University. The aim of this study is to measure and compare the current level of knowledge, attitudes, and practices (KAP) towards COVID-19 pandemic, to correlate the socio-demographic variables to the current level of KAP about COVID-19, and to determine awareness and health behaviors related to the prevention of COVID-19 among the rural and urban dwellers in Sidama region, South Ethiopia; 2020. The purpose of this study is to generate information about the COVID-19 among the adult population who are living in Sidama region. The study may help stakeholders, policy makers, responsible body, and others to take actions based on the finding. The study comprises various socio-demographic and KAP questions. You are chosen to participate in this study by chance. Interview will take not more than 30 minutes.

We assure you that there is no risk or harm in participation of this study. All information will be kept confidentially. Name of a participant will not be written or specified. Your privacy will also be protected and no one shall know your response.

This study benefits you that, you have the right to know about symptoms of COVID-19 pandemic. If you are found to have a risk factor and if you are found to be suspected for COVID-19 pandemic, you will be referred for proper advice and further diagnosis and treatment. There is no incentive or payment for participating in this research. Likewise, findings of the study will show the magnitude KAP among the study population. This in turn will help to design effective and appropriate measure for prevention and control of COVID-19.

You have full right whether or not to participate in this study. You may respond to all questions or you may not answer to the questions you don’t want to or you may quit your participation totally at any time you want. You can ask any questions which is not clear for you.

**2. Informed consent**

As to the information given ahead, participating in this study has no risk. In order to attain the objective of the study, your participation is vital. For this reason we are requesting your free will. You are selected randomly to participate in this study and your name will not be written on this form and the information you give will never be shared to others. Your genuine response to the interviews will be very important for the purpose of the study. You have a right to refuse in responding any question or the entire question at any time you want.

I have read this form or it has been read to me in the language I comprehend and understand all condition stated above.

Are you willing to participate in this study?

Yes □ No □

If “Yes" ...proceed with the interview.

If “No” ...........thank you and end.

Name of the principal investigator: Amanuel Yoseph

Co-investigator: Alemu Tamiso

Amanuel Ejeso

Cell phone Number - +251-915683578 E mail – [amanuelyoseph45@gmail.com](mailto:amanuelyoseph45@gmail.com)

Name of interviewer___________________ Signature__________

Data of interviewer (Ethiopia calendar) _____/_____/_____

Result of interview: 1. Complete 2. Refused 3. Partially complete 4. Respondent not available

Cheeked by supervisor: Name______________ Signature_________ Data___/_____/___

| **Section I: Socio-demographic characteristics** | | | |
| --- | --- | --- | --- |
| No | Questions | \| Response/Alternative  Choices \| \| --- \| | Remark |
| 101 | What is the sex of the respondent? | 1. Male  0. Female |  |
| 102 | What is your age (in completed years)? | __________ years |  |
| 103 | What is your current marital status? (encircle the response) | 1. Single  2. Married  3. Divorced  4. Widowed |  |
| 104 | What is your Religious affiliation? (encircle the response) | 1. Protestant Christian  2. Orthodox Christian  3. Catholic  4. Muslim  5.Other (specify) __________ |  |
| 105 | To which ethnic group do you belong? | \| 1. Sidama  2. Amhara  3. Oromo  4. Gurage  5. Wolayita  88.Other,specify_____ \| \| --- \| |  |
| 106 | What is your highest educational level you have completed? | 1. Cannot read and write  2. Read and write only  3. Primary education(1-8)  4. Secondary education(9-12)  5. Diploma and above |  |
| 107 | What is your family size? (In number) | ________ |  |
| 108 | Place of residence | 1. Rural 2. Urban |  |
| 109 | What is your occupation | 1. Student 2. Merchant 3. Employed 4. Farmer 5. Daily laborer 6. Housewife 7. Others |  |
| 110 | What is your family’s average monthly income? | Approximate_____________ ETB  99. Don’t know |  |

**Section II. Knowledge of respondents regarding COVID-19**

| No | Questions | Response / Alternative Choices | Code |
| --- | --- | --- | --- |
| 201 | Do you hear about COVID-19? | 1. yes  0. no  2. Not sure |  |
| 202 | If yes for above question, from where? | 1.TV  2.Radio  3. Internet, Fb etc  4. Friends/relative  5. Medical staff  6. Others |  |
| 203 | Which of the following is the cause of COVID-19? | 1.Bacteria  2. Virus  3. Fungus  4. Immunodeficiency  5. I don’t know |  |
| 204 | Is COVID-19 a transmissible disease/contagious? | 1. yes  0. no  2. Not sure |  |
| 205 | If yes to question # 203, what ways do you know about its transmission? (more than one answer is possible) | 1.Direct transmission through respiratory droplet  2. Touching surfaces contaminated  with the virus  3. Direct contact with an infected  patient  4. Others  5. I don’t know |  |
| 206 | Do you know the symptoms of COVID-19? | 1. High temp/fever  2. Cough, sore throat, blocked nose  3. Difficulty of breathing  4. Others  5. I don’t know |  |
| 207 | Symptoms appear after which of the following? | 1. <2 days of infection  2. 2 – 5 days  3. 2 – 14 days  4. Others  5. I don’t know |  |
| 208 | Which conditions increase the risk for COVID-19? | 1. Being old egad  2. Individuals with cancer, chronic  respiratory, diabetes  3. Having awakened immune system  4. Others  5. I don’t know |  |
| 209 | Do you think the early diagnosis improves the treatment? | 1. yes  0. no  2. Not sure |  |
| 210 | Is there a treatment for COVID-19? | 1. yes  0. no  2. Not sure |  |
| 211 | Do you think the isolation of the suspected cases is important? | 1. yes  0. no  2. Not sure |  |
| 212 | Is there a vaccine for COVID-19? | 1. yes  0. no |  |
| 213 | Can COVID-19 be prevented? | 1 yes  0. no  2. Not sure |  |
| 214 | If yes to question # 203, what methods do you know to prevent COVID-19? (more than one answer is possible) | 1. Hand washing with soap  2. Maintain social distancing  3.Wear cloth face mask  4.Using alcohol or sanitizer  5. Disinfecting frequently touched surface and objects  6. Avoid touching noses, mouse, and eyes with unwashed hands  7. Stay at home  8.Other, specify_______________ |  |
| 215 | Do you think that health education can help to prevent the disease? | 1 yes  0. no  2. Not sure |  |

**Section III. Attitude of respondents regarding COVID-19**

| No | Questions | Response / Alternative Choices | Code |
| --- | --- | --- | --- |
| 301 | Do you think that the disease is dangerous? | 1. yes  0. no  2. Not sure |  |
| 302 | Are you worried about one of your family members can get infection? | 1. yes  0. no  2. Not sure |  |
| 303 | Are you afraid to go to common places in order to avoid infection? | 1. yes  0. no  2. Not sure |  |
| 304 | If you take precautions, can the COVID-19 infection be prevented? | 1. yes  0. no  2. Not sure |  |
| 305 | Should you know information about COVID-19? | 1. yes  0. no  2. Not sure |  |
| 306 | If you know that the animals are sources for the transmission of COVID-19, would you consume raw animals’ milk or meat? | 1. yes  0. no  2. Not sure |  |
| 307 | If there is a vaccine, would you take it? | 1. yes  0. no  2. Not sure |  |
| 308 | Can COVID-19 about infection be cured? | 1. yes  0. no |  |
| 309 | Is the available information about COVID-19 in Ethiopia sufficient? | 1 yes  0. no  2. Not sure |  |
| 310 | Are the protective measures sufficient for prevention? | 1 yes  0. no  2. Not sure |  |
| 311 | Is there negative effect of infection on Ethiopia economy? | 1 yes  0. no  2. Not sure |  |
| 312 | Does the government institutions able to control the pandemic? | 1. yes  2. no  2. Not sure |  |
| 313 | Do you think yourself at risk? | 1. yes  2. no  2. Not sure |  |
| 214 | If you have one of the symptoms of the disease do you go to the Doctor? | 1. yes  2. no  2. Not sure |  |
| 315 | If you have COVID-19 symptoms, do you avoid normal activities? | 1. yes  2. no  2. Not sure |  |
| 316 | Do you avoid contact with infected case? | 1. yes  2. no  2. Not sure |  |
| 317 | Do you take safety precautions and prevention? | 1. yes  2. no  2. Not sure |  |
| 318 | Do you think the early diagnosis improves the treatment? | 1. yes  0. no  2. Not sure |  |
| 319 | Do you think the isolation of the suspected cases is important? | 1. yes  0. no  2. Not sure |  |
| 320 | Do you think that health education can help to prevent the disease? | 1 yes  0. no  2. Not sure |  |

**Section III. Practices of respondents regarding COVID-19**

| No | Questions | Response / Alternative Choices | Code |
| --- | --- | --- | --- |
| 401 | Which of the following precautions do you take to prevent Coronavirus infection? | 1. I wash hands often. 2. I avoid touching the eyes, nose and mouth. 3. I throw the tissue in the trash after I use. 4. I use masking to cover my nose in crowded places 5. Stay at home 6. Maintaining social distance 7. Disinfecting frequently touched surface and objects 8. All of the above. |  |
| 402 | Which of the following is the reason for getting vaccination (if present)? | 1. Required for my job. 2. Doctor/HCP advised me to do it. 3. Because of my age. 4. It is free |  |
