# Supplementary material for: Knowledge, attitudes, and practices related to COVID-19 pandemic among adult population in Sidama Regional State, Southern Ethiopia: A community based cross-sectional study
Source: PLoS One. 2021 Jan 29;16(1):e0246283. doi: 10.1371/journal.pone.0246283 (PMC7846007; doi:10.1371/journal.pone.0246283)
Supplement: S2 File — (DOCX) [file pone.0246283.s002.docx]

HAWAASI YUNIVERSTE XAGISSATENNA FAYYIMMATE KOLEJE,DAGATE FAYYIMMA ROSI MINE

Xa’muwa fillonihu dagaate xaphphoomunni nonsa EGEENO,LAOOSHENNA ROSICHONSHA xinxalinaanni kooronu/kovidi_19 dhibbi dafira sidaamu qoqqowi gido gammareeyye calla xa’minanni xa’muwwa

taje uynanni qolla

keereho !! su’mi’ya_______________________________yinanie . Ani taje dagaaniwinni gamba assamora dawoomo/ma ,konni haaru kooronu /kovid dhibbi dafira ,hawaasi yuniversite xagissatenna fayyimmate koleejera woroonni. Tene xinxaalloti gulcho amado,tenne yannara kalqeete dhibbi qaru qarri, kooronu/kovid dhibbi dafira dagaate egeeno,laoshshenna rosichichu derra bikkitenna hewississate . Koni’rano daganna dagaamu amado la’nohunni xa nonsa eggeno laoshenna rosichinsa derrana hattono koronu dhibbi dafira nonsa baqqinaate affateeti. Woleno akaatu ledo no gargadhaate amanyoote badiyyetena quchumaho hedhaano Sidaamu qoqqowi gido no tessanora affate, hawassa, itiyophiya, 2012. Tene xinxaalote gulcho amado,mashalaaqe afi’rate koronu /kovid dhibbi dafira hedhaano Sidaamu gido hedhaano tessanora. Tini xinxaalo lowo geshsha ka’litannohu rorenkanni hajo la’anonsa bissara,hajaajo uytano bissara,animaate masaagano bissararanna xinxallote afi’nonni gumunni lonsanni lossira fakkana rosiicho uyitanno yine hendaanni. Xa’muwaate qara amado daganna dagoomu akaata,hattono dagaate nonsa eggeno laoshshena rosiichu aana illachishanno . Konni dafira ati tene xinxaalora beqqancho ikkite doraamota/to.tene xa’muwa xa’maate 30 daqiqqa calla ane ledo keshshineemo. Xa’mamota/to dafira mitte gawajjo woy utto dihedhaanohe. Atewinni afi’neemo taje foojo /kawajjo lowontanni huluulo noki gede agareemo yanni qaale e’emo. Ate su’ma diboressemo/ma,dawaro qolatoota ayino foojokki agaraanno gede duure kuleemo. Xinxaalote horo,koronu dhibbi malaatassi affate ,xiiso hedhuro naffa xaginsanninna amalaanni .Tene xinxallora mittoreno woxe dibaantanni harunsooto/ta dafira. Wole kayinni xinxaalote guma magesha daga koronu dhibbi dafira no derra affateeti.xinxaalote beqqancho diikkimo/ma yaate womunni wo’ma qossokki aggaraantinoote. Xa’mo hedhuhero xa’ma dandaato/to.

sumuu yaato ? 1. Ee 2. De’ni ee yiiro xa’moniwa sai de’ni yiiro urrissi.

Xinxalaano 1kkihu ; Alemu Xa’misoho

2kkihu; Amanueli Yoosefiho

bilibilla 0915683578 email [amanuelyoseph45@gmail.com](mailto:amanuelyoseph45@gmail.com)

xa’manchu su’ma_______________________________________________malaate___________________

xa’minanni barra________/___________/________________

buuxxinohu qorqoraanchu su’ma___________________malaate______________________barra______/_________/____

Gaamo I: Daganna dagamu akaata illachishshano xa’muwwa**.**

| 101 | Dawaranchu koo/tee akata? | 1. koo  2. tee |  |
| --- | --- | --- | --- |
| 102 | Dirooki | --------------- |  |
| 103 | Adhaamate akata? | 1. diadhomo/diadhamoma  2. adhamoma/mo  3. adhame tiromo/ma  4. galte’ya reyno. |  |
| 104 | Amanokki hitteneti? | 1. kirstanaho  2. ortodokisete  3. katolikete  4. islaamsaho  5.wole xawisi __________ |  |
| 105 | Hittene daga gido gamammato? | \| 1. Sidaama  2. Amara  3. Oromo  4. Gurage  5. Wolayita  88.wole xawisi_____ \| \| --- \| |  |
| 106 | Rosiki derri mageeshshsaati? | 1.nabawannaborressa didandeemo/ma.  2.nabawanna borressa calla dandema/mo.  3. umi derra guduumo/ma(1-8)  4. layinki derra guduumo(9-12)  5. 12+3(Diplomana hakkuni aleeni rosoma/mo |  |
| 107 | Maateki kiiro mageshshaati? | ________ (kiiroteni wori) |  |
| 108 | Teesoki | 1. Badiyye 2. Quchuuma |  |
| 109 | Loosikki qeecha | 1. Rosanchoho 2. dadalanchoho 3. mangistete loasasiincho 4. baato losi’re galooma/ma 5. barru losasiincho 6. minu amaati. 7. Wole xawisi____________ |  |
| 110 | Aganunni mereerima eokki mageeshshaati? | Rekkotenni itiophiyyu birrinni wori foqa darga_____________  99. Diaffomo/ma |  |

**GAAMO II: KOORONU DHIBBI DAFIRA DAGAATE NONSA EGEENO KEENANNI XA’MUWWA**

| A.K | Xa’muwwa | Dawaaro dorranni dorsha | kode |
| --- | --- | --- | --- |
| 201 | Koorunu vayrese dhibbi/kovid_19 dafira maciishite egenota/to? | 1.Ee  2. De’ni  3.dibuuxomo/ma |  |
| 202 | 201kki dawaaro ee yitoro/yitaro hiikinni maciishshito/ta? | 1.televizhinetenni  2.Radonoteenni  3.tuqqu xaadinni.  4. jaala’yawinni/fiixi’yawinni.  5. wole xawisi._________________ |  |
| 203 | Hittenne shiimadda lubuwanni tarawaano kuni koorunu dhibbi/kovid-19? | 1.bakiteriyunni  2. vayresetenni  3. fangesetenni  4. dhibba gargartano biisinke sella ajjatenni  5. Diaffomo/ma |  |
| 204 | Kuni kooronu dhibbi manchuni mancho taraawanno yite heedaato/ta? | 1. Ee  2. de’ni  3. dibuuxumo/ma |  |
| 205 | 204kki xa’mora dawaarokki ee yitoro/taro hittonni tarawaano yite heedaata/to?(mituni aleenni dorra dandinanni) | 1.xadoote fulaano,foolate amanyooti widoonni sandidooninna woy anjinni.  2. illete leltanokki vayiresenni dafaamino bayicho amadaatenni.  3. kooronu dhibbinni dafaamino mancho xaadotenni xaadaatenni.  4. Wole xawisi________________  5. diaffoma/ma |  |
| 206 | Koorunu/kovid-19 dhibbi malaata affoto/ta? | 1.daana saino bisu ibbilli  2. buusanno, affuu garru madi’ra, sano follanikki gede fuga.  3. foole lamewa taysa.  4. wole xawisi_________________  5. diaffoma/mo |  |
| 207 | Kooronu/kovid-19 dhibbi amadino manchi meu barrinniniti malaatu leelanohu? | 1. lamu barrinni ajjinno barri gido.  2. 2 – 5 barri gido  3. 2 – 14 barri gido  4. wole xawisi___________  5.diaffomo/ma |  |
| 208 | Rorenkanni reqqecci yino manni hikkuriuuti konni kooronu/kovid-19 dhibbira? | 1.dirrunni luuphi yaa(geedha)  2.kanserete(balaamu) dhibbi no manchira,keeshino foolate amanyooti dhibbi no manira , mundensa gido suukareete dhibbi no manira  3. wolu dhibbinni daafurinno manira.  4. wole xawisi_______________  5. diaffoma/mo. |  |
| 209 | Muleeni fayyimmate ogeeyyenni bunxe xaginsiro kooronu dhibbinni huraano yite heedaato/ta? | 1. Ee  2. De’ni  3. dibuuxoma/mo |  |
| 210 | Kooronu dhibbira/kovid-19 xagichu noosi? | 1. Ee  2. de’ni  3. dibuuxomo/ma |  |
| 211 | kooronu dhibbinni huluulamonni mancho mannuni xaada hoola danchaate yite heedato/ta? | 1. ee  2. de’ni  3. dibuuxomo/ma |  |
| 212 | Kooronu dhibbira kitibaatu noosi? | 1. ee  2. de’ni  3. dibuuxomo/ma |  |
| 213 | Kooronu dhibbi amadikkinni balanxe gargadha dandinanni? | 1 ee  2. de’ni  3. dibuuxomo/ma |  |
| 214 | 2013kki xa’mora dawaro ee yituro/toro Kooronu dhibba hittene dogonni gardadhinaanni?(mittuni aleeni dorra dandinanni.) | 1.saamununni anga hayishira  2. manuwinni faanfe woy xeritirra.  3affoona sano diwanoricho/maske horonsira.  4.alkole woy angate coyimma agadhinaanirinni mule muleni horonsira.  5.daaffamiino uduune alkoletenni woy coimma uyinannirinni coyimmasi agarate .  6. sanno,affoona ille hayishi’nonikki anganni amaaxa hogaatenni. agaadhaatenni.  7.mininni fula hogaatenni.  8.wole, xawisi_______________ |  |
| 215 | Fayyimmate roso dagaate rosiissa kooronu dhibbini gargatanno yite heedato/ta? | 1 ee  2. de’ni  3. dibuuxomo/ma |  |

**Gaamo III: kooronu/kovid-19) dhibbi dafira dagate laooshe kenanni xa’muwwa.**

| A.K | XA’MUWA | Dorranni doorsha. | kode |
| --- | --- | --- | --- |
| 301 | Kunni kooronu dhibbi lowontanni hantalaamoho/ gawaajanno) yite hedaato/ta? | 1. ee  2. de’ni  3. dibuuxomo/ma. |  |
| 302 | Matekki gido kooronu dhibbinni amadaama dandaano/danditanno yite yaadatohu/tahu no? | 1. ee  2. de’ni  3. dibuuxomo/ma |  |
| 303 | Gamba yine mittenni ha’nani baycho ma’late koorunu dhibbinni daffamemo/ma yite waajjato/ta? | 1.ee  2.de’ni  3. dibuuxomo/ma |  |
| 304 | Kooronu dhibbinni daffamatewinni balanxe qorophiniro gantanni yite hedaato/ta? | 1. ee  2.de’ni  3.dibuuxomo/ma . |  |
| 305 | Kooronu dhibbi dafira mashshalaaqe hedhanohe? | 1. ee  2. de’ni  3. dibuuxomo/ma |  |
| 306 | Koorunu dhibbi tarawoora kaimu sadaate yiniro,maala woy ado saga’lato/ta? | 1. ee  2. de’ni  3.de’ni |  |
| 307 | Kooronu dhibbira kitiwaatu heriro,xagicho adhato/ta? | 1.ee  2. de’ni  3. dibuuxomo/ma |  |
| 308 | Kooronu dhibbinni hurranni yite hedaato/to? | 1. ee  2. de’ni  3. dibuuxomo/ma |  |
| 309 | Kooronu dhibbi dafira gobbankera uyinonniti ikkado tajeeti yite hedaato/ta? | 1 ee  2. de’ni  3. dibuuxomo/ma |  |
| 310 | Koorunu dhibbi dafira affi’nonni mashalaqqe gargadhaatenna qorophphaate ikkadote yite heedaato/ta? | 1 ee  2. de’ni  3. dibuuxomo/ma |  |
| 311 | Itiyophphiyu minji kooronu dhibbinni gawaajamanno yite hedaato/ta? | 1 ee  2. de’ni  3. dibuuxumo/ma |  |
| 312 | Konne taraawanno dhibba mangitete urrinshsha gargara danditanno yite hedaato/ta? | 1. ee  2. de’ni  3. dibuuxoma/ma |  |
| 313 | Ati umikinni kooronu dhibbira reqqecci yototta/ta affooto/ta? | 1. ee  2. de’ni  3. dibuuxoma/mo |  |
| 314 | Kooronu dhibbi malaati mitu naffa lelihero hakkimete minira ha’raato/ta? | 1. ee  2. de’ni  3. dibuuxoma/mo |  |
| 315 | Konni haaru dhibbi kooronu malaati lelihero lossokki aguraato/ta? | 1.ee  2. de’ni  3. dibuuxoma/mo |  |
| 316 | Kooronu dhibbinni dafamino mancho balaaxe gargardhaato/ta? | 1. ee  2. de’ni  3. dibuuxoma/mo |  |
| 317 | Kooronu dhibbi qo’rophphorana gargadhara balaaxe qaafo adhooto/ta? | 1. ee  2. de’ni  3. dibuuxoma/mo |  |

**Gaamo IV: KOORUNU VAYRESE DHIBBIRA DAGOOMU AFIRINO ROSIICHCHO KENAANNI XA’MUWWA**

| 401 | Koorunu dhibbi taraawo gargadhaate adhoto/ta qorophpho hitteneeti? | 1. Anga duucha woyte hashiraate 2. Illenke ,sanonkenna affoonke amaaxa hogaateenni. 3. Cooimma agadhinaanirinni horonsi’re kaumoro mulaawa oolle hunaatenni. 4. Mannu tuddino darga maruumoro affoona sano tua’anorinni dhiiphaatenni. 5. Mininni fula hogaatenni. 6. Mannu xertinye agadhaatenni. 7. Horonsi’neemo uduunicho coimma mule mule agaratenni. 8. Aleeni yini’ri balunku dawaaroote. |  |
| --- | --- | --- | --- |
| 402 | Kooronu kitiwaati herri ikkiro, afi’raate ,korkatikki maati? | 1. Loosi’yara hasireeti. 2. Fayyimmate ogeeyye amaalenniniti. 3. Korkatunno,dirri’ya derriniti. 4. Kitiwaate baatoshiweelo uyinanni dafiraati. |  |
